# Supplementary material for: Inhibiting NLRP3 Inflammasome Activation by CY-09 Helps to Restore Cerebral Glucose Metabolism in 3×Tg-AD Mice
Source: Antioxidants (Basel). 2023 Mar 15;12(3):722. doi: 10.3390/antiox12030722 (PMC10045645; doi:10.3390/antiox12030722)
Supplement: Supplementary file 1 [file antioxidants-12-00722-s001.zip › antioxidants-2223275-supplementary.pdf]

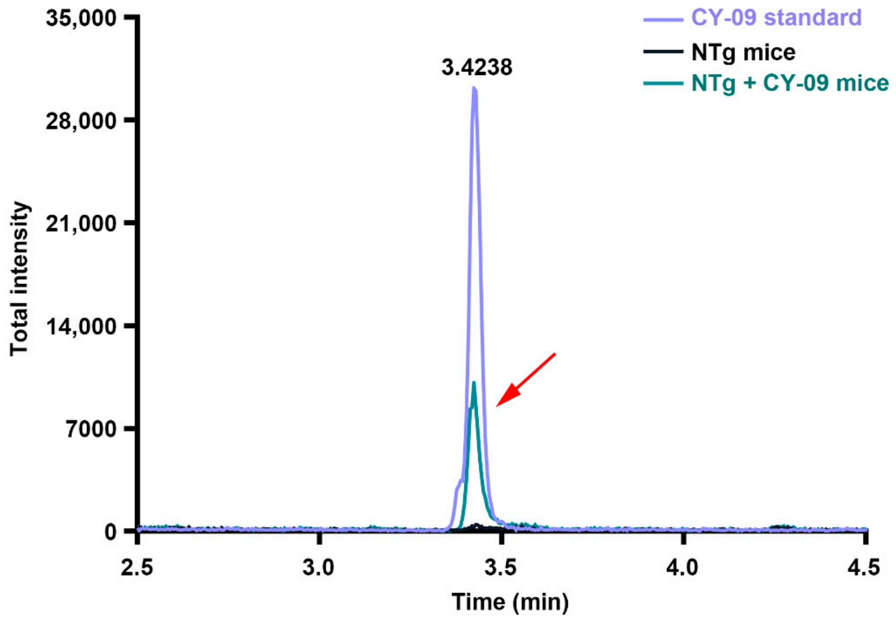

Figure S1. Content of CY-09 in NTg and NTg + CY-09 mice brains.

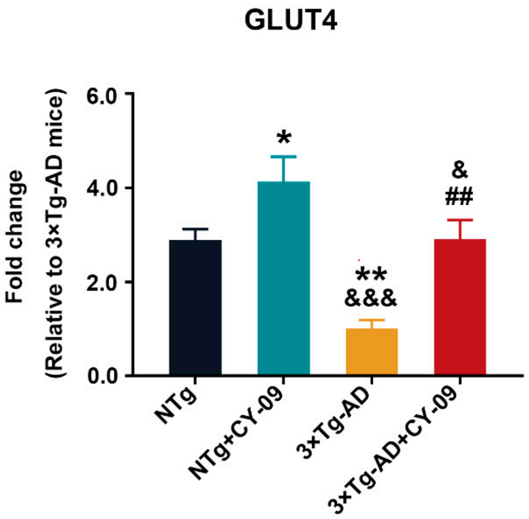

Figure S2. Fluorescence intensity of GLUT4 in NTg, NTg + CY-09, 3×Tg-AD and 3×Tg-AD + CY-09 mice (n=6, mean ± SD, one-way ANOVA and Bonferroni post hoc test; * $p < 0.05$, ** $p < 0.01$ vs. NTg mice, $^{\&}$ $p < 0.05$, $^{\&\&\&}$ $p < 0.001$ vs. NTg + CY-09 mice, ## $p < 0.01$ vs. 3×Tg-AD mice).

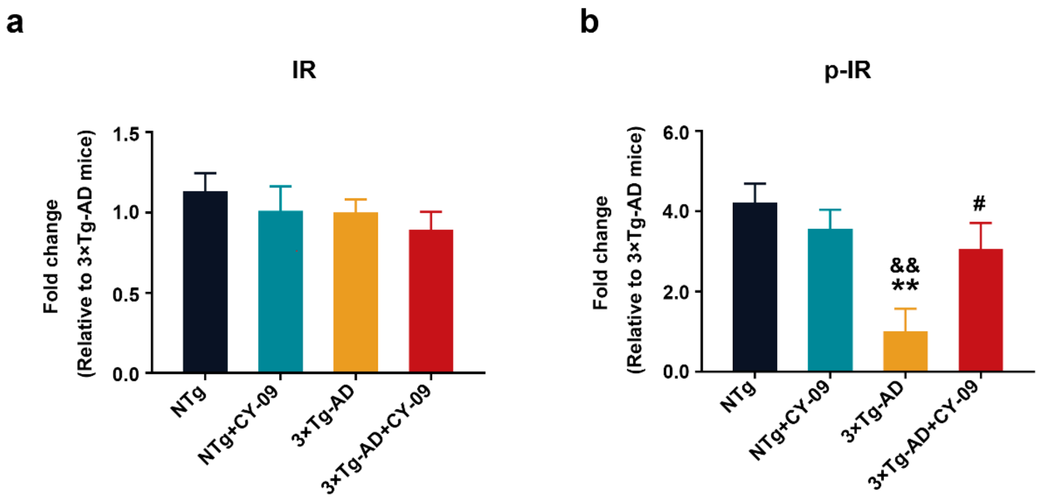

Figure S3. Fluorescence intensity of (**a**) IR and (**b**) p-IR in NTg, NTg + CY-09, 3×Tg-AD and 3×Tg-AD + CY-09 mice (n=6, mean ± SD, one-way ANOVA and Bonferroni post hoc test; ** $p < 0.01$ vs. NTg mice, && $p < 0.01$ vs. NTg + CY-09 mice, # $p < 0.05$ vs. 3×Tg-AD mice).

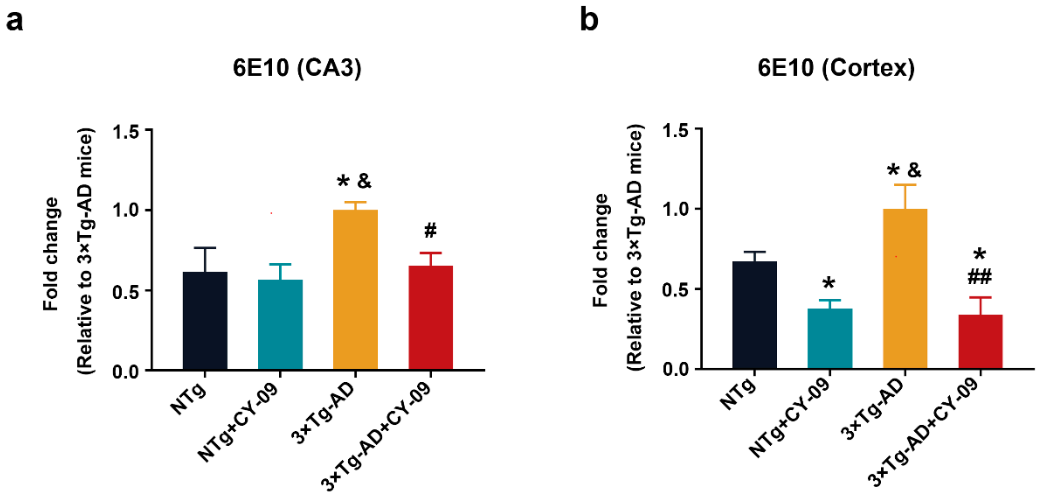

Figure S4. Fluorescence intensity of Aβ (6E10) in (**a**) CA3 region and (**b**) cortex of NTg, NTg + CY-09, 3×Tg-AD and 3×Tg-AD + CY-09 mice (n=6, mean ± SD, one-way ANOVA and Bonferroni post hoc test; * $p < 0.05$ vs. NTg mice, & $p < 0.05$ vs. NTg + CY-09 mice, # $p < 0.05$, ## $p < 0.01$ vs. 3×Tg-AD mice).

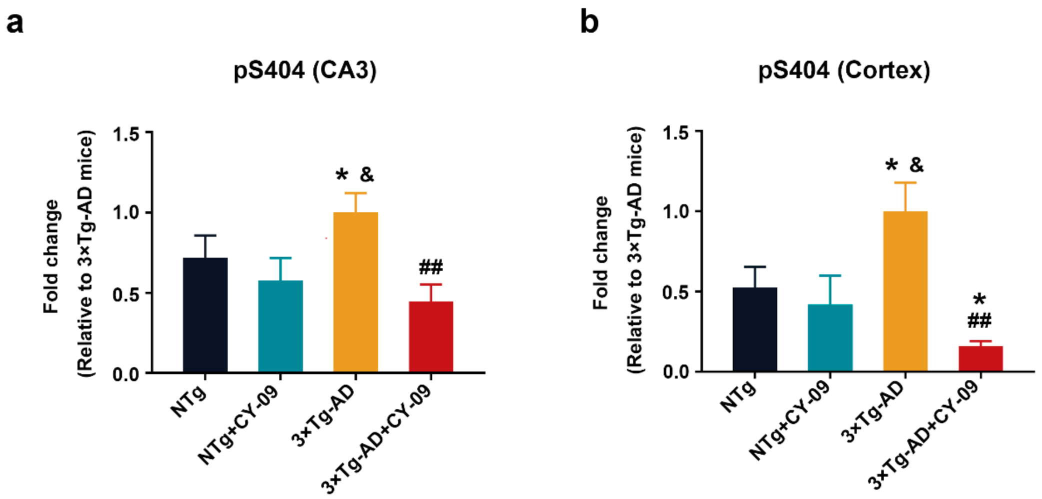

Figure S5. Fluorescence intensity of p-tau-S404 in (**a**) CA3 region and (**b**) cortex of NTg, NTg + CY-09, 3×Tg-AD and 3×Tg-AD + CY-09 mice (n=6, mean ± SD, one-way ANOVA and Bonferroni post hoc test; * $p < 0.05$ vs. NTg mice, $^{\&}$ $p < 0.05$ vs. NTg + CY-09 mice, ## $p < 0.01$ vs. 3×Tg-AD mice).
